# Trait anxiety is related to Nx4’s efficacy on stress-induced changes in amygdala-centered resting state functional connectivity: a placebo-controlled cross-over trial in mildly to moderately stressed healthy volunteers

**DOI:** 10.1186/s12868-022-00754-4

**Published:** 2022-11-24

**Authors:** Melanni Nanni-Zepeda, Sarah Alizadeh, Tara Chand, Vanessa Kasties, Yan Fan, Johan van der Meer, Luisa Herrmann, Johannes C. Vester, Myron Schulz, Britta Naschold, Martin Walter

**Affiliations:** 1 Department of Psychiatry and Psychotherapy, Jena University Hospital, Philosophenweg 3, 07743 Jena, Germany; 2 Department of Psychiatry and Psychotherapy, University of Tübingen, Calwerstraße 14, 72076 Tübingen, Germany; 3 Leibniz Research Centre for Working Environment and Human Factors, Ardeystraße 67, 44139 Dortmund, Germany; 4 Department of Radiology and Nuclear Medicine, Amsterdam University Medical Center, Meibergdreef 9, 1105 AZ Amsterdam, Netherlands; 5 idv Data Analysis and Study Planning, Tassilostraße 6, 82131 Gauting, Germany; 6 Biologische Heilmittel Heel GmbH, Dr.-Reckeweg-Str. 2-4, 76532 Baden-Baden, Germany

**Keywords:** Stress, Anxiety, FMRI, Amygdala Complementary therapies, Neurexan

## Abstract

**Background:**

The multicomponent drug Neurexan (Nx4) was shown to reduce the neural stress network activation. We now investigated its effects on stress-induced resting state functional connectivity (RSFC) in dependence of trait anxiety (TA), an acknowledged vulnerability factor for stress-induced psychopathologies.

**Methods:**

Nx4 was tested in a randomized placebo-controlled crossover trial. Resting state fMRI scans were performed before and after a psychosocial stress task and exploratively analyzed for amygdala centered RSFC. Effects of Nx4 on stress-induced RSFC changes were evaluated and correlated to TA levels. A subgroup analysis based on TA scores was performed.

**Results:**

Multiple linear regression analysis revealed a significant correlation between TA and Nx4 effect on stress-induced RSFC changes between right amygdala and pregenual anterior cingulate cortex (pgACC) and ventro-medial prefrontal cortex (vmPFC). For participants with above average TA, a significant amelioration of the stress-induced RSFC changes was observed.

**Conclusions:**

The data add evidence to the hypothesis that Nx4’s clinical efficacy is based on a dampened activation of the neural stress network, with a greater neural response in subjects with anxious personality traits. Further studies assessing clinically relevant outcome measures in parallel to fMRI are encouraged to evaluate the real-world benefit of Nx4.

*Trial registration* NCT02602275.

**Supplementary Information:**

The online version contains supplementary material available at 10.1186/s12868-022-00754-4.

## Background

### Trait anxiety shapes the stress response

Psychosocial stress can generate temporary discomfort and long-term health consequences [1, 2]. The subjective experience of stress, as well as the response to stress is different in every individual. Several studies have investigated personality traits as important components that determine how individuals approach and cope with stress and as important vulnerability factors for stress-induced psychopathologies [3–6]. Trait Anxiety (TA), defined as the tendency of individuals to experience frequent and high-intensity anxiety and worry in the face of stressful situations [7], is one of the most relevant personality traits that shape the response to a wide variety of stressors [8]. TA plays a significant role in interindividual differences in stress vulnerability. Particularly, high TA favors a more reactive physiological stress response [9] instead of actively coping with stress. A strong relationship between TA and day-to-day stress responsiveness in terms of subjective, cognitive, behavioral, and physiological responses to social stress has recently been postulated [10].

### The amygdala plays an important role in psychosocial stress and anxiety

For the neural basis of stress and anxiety, it has been demonstrated extensively that specific areas in the brain are involved in both stress and anxiety processes, including the amygdala, prefrontal cortex, cingulate cortex, hypothalamus and brainstem nuclei [11–13]. It is well known that the amygdala and its widespread cortical and subcortical connections play an important role in psychosocial stress and anxiety. The amygdala is associated with emotional and social processing [14–16], and with the initiation of physiological responses to fear and stress, including activation of the sympathetic nervous system and the hypothalamic–pituitary–adrenal (HPA) axis [17–19]. Several studies in healthy cohorts have demonstrated a relationship between high TA and amygdala dysregulation during the processing of aversive and neutral stimuli or negative emotion processing (angry faces), indicating an increased amygdala activation in anxiety-prone subjects [20–23].

### Stress induces changes in resting state functional connectivity of the amygdala

Even in the resting state (RS) of the brain, in the absence of an externally prompted task, previously perceived stress and anxiety affect the intrinsic activity of the amygdala and its functional connectivity (FC). Studies that investigated spontaneous brain activity at rest, immediately after experimental stress induction, found that the organization of the amygdala’s FC network at rest is perturbed by acute stress and during affective recovery [13, 24–27]. Similarly, aberrant amygdala resting-state functional connectivity (RSFC) was found in patients with Generalized Anxiety Disorder [28], and other stress-related psychiatric conditions like Major Depressive Disorder [29–31], Post-Traumatic Stress Disorder [32–35] and anxiety disorders [33, 36].

### Nx4 showed stress-relieving effects on a behavioral and on a neural level

Nx4 (Neurexan; Heel GmbH, Baden-Baden, Germany) is a natural medicinal product composed of herbal extracts of oat, coffee, passionflower, and a mineral salt at low, ponderable concentrations. Stress-relieving effects of Nx4 were observed in two observational studies for the stress-related conditions insomnia [37] and nervous restlessness [38]. In a randomized controlled trial in healthy subjects, Nx4 modulated the peripheral physiological stress response to an acute laboratory stress task, particularly by reducing salivary cortisol and plasma adrenaline release [39]. Recent analyses of the NEURIM study revealed that Nx4 ameliorated stress induced changes of heart rate variability and alpha and theta electroencephalography (EEG) oscillations [40], reduced the susceptibility to distraction in attention modulation task [41], reduced the amygdala activation in response to negative emotional stimuli [42], attenuated the activation of the anterior cingulate cortex in response to psychosocial stress induction [43], modulated task free RSFC of amygdala [44], and improved vigilance regulation in RS after stress induction [45].

### Objective of this study: effect of Nx4 on amygdala RSFC after stress induction is a function of TA

The NEURIM trial exploratively assessed the effect of Nx4 on the stress response by various outcome measures, i.e. functional magnetic resonance imaging (fMRI), EEG, blood and saliva stress biomarkers, and patient-reported outcomes. In the analysis described in this manuscript, we investigated the effect of TA on the functional network of the amygdala at rest after a single dose of Nx4 followed by stress induction. Given the previously described effects of Nx4, the central role of the amygdala in stress response, and the role of TA as a vulnerability factor for stress related symptoms, we hypothesized that the effect of Nx4 on stress-induced changes of the amygdala RSFC is correlated on the individual TA scores of the study participants. Based on this correlation, we hypothesized further that Nx4 significantly reduces stress-induced changes of the amygdala RSFC in a subgroup of patients with an above average TA.

## Methods

### Overall trial design

**Fig. 1:**
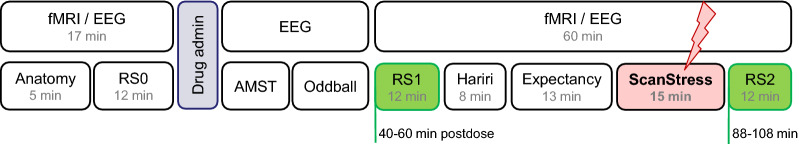
Study flow of the NEURIM trial. On each of the two study days, a structural MRI scan and a resting state (RS) measurement were conducted during a simultaneous EEG/fMRI scan session. After administering a single dose (three tablets) of Nx4 or placebo, two computerized tests, the Attention Modulation by Salience Task (AMST) and an auditory oddball task, were performed while EEG data were acquired. A second EEG/fMRI scan session was conducted, starting 40 to 60 minutes after dosing, including an initial RS session (RS1) followed by the Hariri emotional face-matching task, an expectancy task, and the ScanSTRESS paradigm as well as another resting-state session (RS2). This publication focuses on the fMRI data from RS1 (pre-stress) and RS2 (post-stress) marked in green

Neuronal correlates of Nx4 were evaluated in an exploratory clinical trial (NEURIM; ClinicalTrials.gov identifier: NCT02602275; registered 28/10/2015) whose first primary endpoint has been published previously [42]. In this randomized, placebo-controlled, double-blind, two-period, two-treatment crossover trial with 1:1 randomization of the two treatment sequences, Nx4-Placebo and Placebo-Nx4, a total of 40 participants were included at a single site at the Clinical Affective Neuroimaging Laboratory (CANLAB), Magdeburg, Germany. Study participants were healthy males, aged 31 to 59 years, with mild to moderate chronic stress defined by a Trier Inventory for Chronic Stress (Short Screening Scale for Chronic Stress; TICS-SCSS) between $\ge \ 9$ and $\le \ 36$, as well as a Perceived Stress Scale (PSS) of $>9$. Participants received a single dose of three tablets Nx4 or placebo on each of the two study days (Day 1 and Day 2) with a washout period of 7 to 35 days in between. On each of the two study days, several EEG, fMRI and psychosocial tests were performed as given in Fig. 1. This publication describes the analysis of the RS-fMRI data acquired shortly after dosing (RS1) and after a psychosocial stress induction (RS2). To minimize any confounding effects of circadian rhythm, the RS-fMRI measurements were performed at almost the same time of the day, in the afternoon.

### Safety and numbers of participants analyzed

**Fig. 2:**
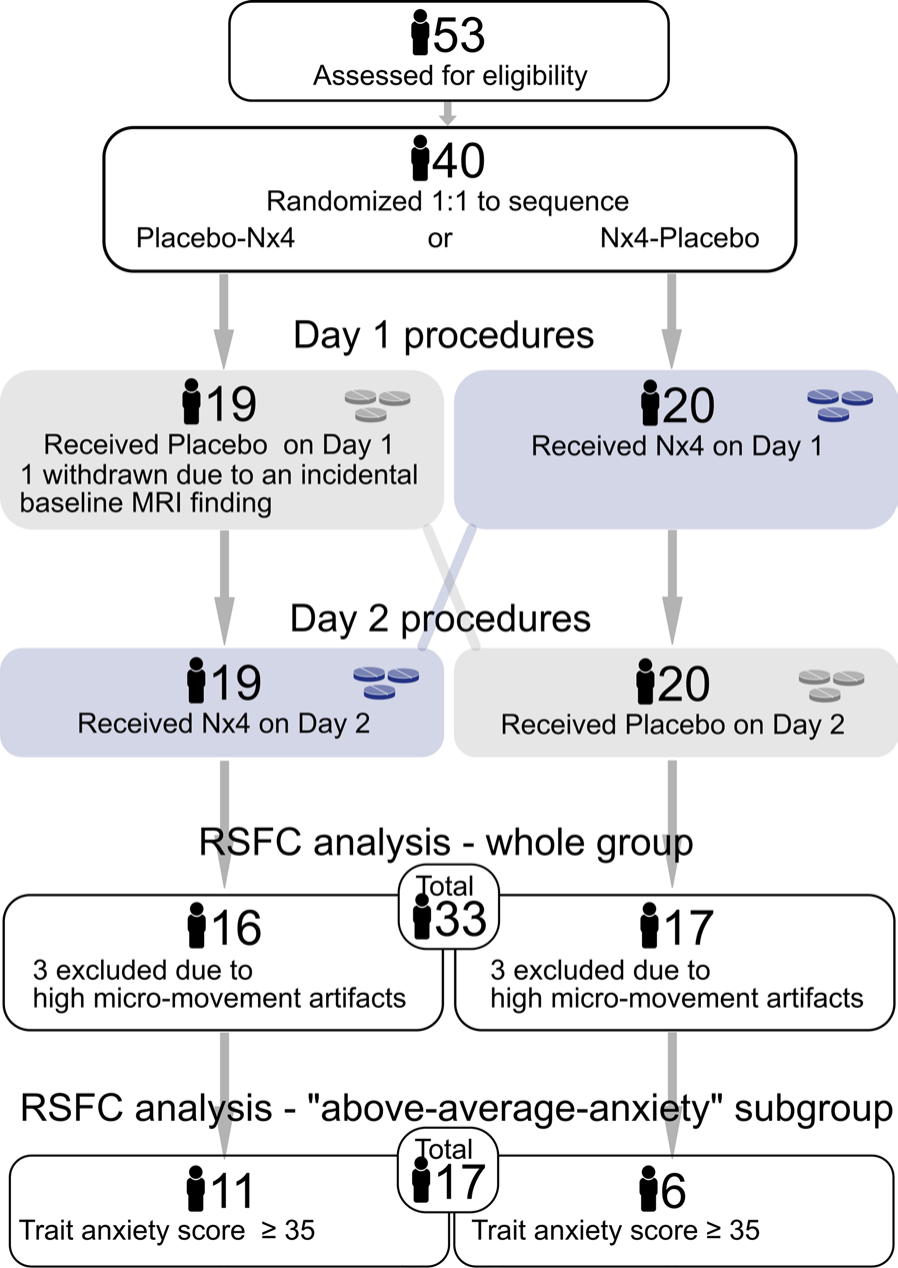
Patient Flow. Number of participants in the two sequences, placebo first and Nx4 first, analyzed for stress-induced changes in resting state functional connectivity (RSFC) from pre-stress RS1 to post-stress RS2. The final sample in the whole group analysis was 33 and in the above average trait anxiety (TA) subgroup analysis 17 participants

As described previously, the single dose treatment with three tablets of Nx4 was considered safe and well tolerated [42]. From the 53 screened participants, a total number of 40 healthy males were eligible and included. Twenty participants were randomly assigned to each of the two treatment sequences, placebo first or Nx4 first (Fig. 2). Participants were in the age range of 31 to 59 years and had a mild to moderate level of stress. One participant of the placebo first sequence dropped out of the trial due to an incidental baseline MRI finding before drug administration. Out of the 39 participants completing the trial, 33 were included in the RSFC analysis (17 participants receiving Nx4 first and 16 participants receiving placebo first). Six participants (3 in each sequence) were excluded due to motion artifacts. From the 33 participants in the RSFC analysis (Age: 43.19.7, TA: 36.17.4), 17 were included in the above average TA subgroup (Age: 40.69.4, TA: 41.16.5), defined by a baseline $TA \ge \ 35$. Six were in the Nx4 first, 11 in the placebo first sequence. Number of participants, age, TA, TICS, and PSS scores are given in table 1.

**Table 1:** Demographics. Summary statistics for number of participants (N), Age in years, Trait Anxiety (TA), Trier Inventory for Chronic Stress (TICS) and perceived stress scale (PSS) are shown for the whole group as well as high and low anxiety subgroups

	N	Age	TA	TICS	PSS
Whole group	33	43.19.7	36.17.4	15.75.7	14.73.7
High anxiety	17	40.69.4	41.16.5	17.16.3	15.34.6
Low anxiety	16	44.210.2	30.73.7	14.14.6	14.02.3

The low and high anxiety subgroups are defined based on the median of the TA score

### Anxiety assessment

**Table 2:** Significant cluster with an correlation between Nx4 effect on stress-induced changes of functional connectivity to the right amygdala associated with trait anxiety scores

Region	Cluster-level		Peak-level	MNI coordinates		
	Size	FDR-corr	t-value	x	y	z
pgACC/vmPFC	65413	0.005	4.25	0	51	− 15
			4.18	6	39	3
			3.84	− 6	45	− 9

*MNI* Montréal Neurological Institute, *pgACC* pregenual anterior cingulate cortex, *vmPFC* ventro-medial prefrontal cortex, *FDR-corr* False Discovery Rate corrected

The German version of the State-Trait-Anxiety Inventory [46] was used to assess TA. TA characteristics can be defined as feelings of stress, worry, discomfort, etc. that one experiences on a day-to-day basis [47]. Answers to the 20 items questionnaire are given in a 4-point rating scale ranging from 1 = “almost never” to 4 = “almost always”. Some TA questions relate to the absence of anxiety and are reversely coded. Score range is 20–80 and higher scores indicate a higher anxiety. The TA was assessed once, at the screening visit, approximately three to seven days before Day 1.

### Psychosocial stress induction

In this publication, we focused on the effects of the psychosocial stress induction, elicited by the ScanSTRESS task [42, 48], present in the post-stress RS2 compared to pre-stress RS1. The ScanSTRESS paradigm is an fMRI compatible adaptation of the Trier Social Stress Test, and applies several dimensions of stress, including pressure to perform, time pressure, forced failure, social-evaluative threat, uncontrollability and unpredictability [48]. It was composed of two runs, with alternating blocks of control and stress conditions of serial subtraction tasks and mental rotations. Control blocks did not contain any social evaluative feedback, time pressure or difficult questions whereas during stress blocks, participants were pushed for time, and two experimenters in professional attire explicitly showed their dissatisfaction with the correctness and speed of the answers via video stream. Task speed and difficulty were automatically adapted to the individual performance, ensuring that the participants were unable to meet the expectations. Between the two runs of the task, participants were interrupted and given extensive, disapproving verbal feedback (see also Additional file 1: Fig S1). The effects of the stress task in this trial on stress network activation during the task as well as on vigilance state and heart rate variability at rest after the task are described elsewhere [40, 42, 45]. In this manuscript, we assessed stress task induced changes in amygdala RSFC from RS1 to RS2 and their relation to TA Additional file 2: Fig S2.

### fMRI data acquisition

A Philips 3T scanner was used for fMRI data acquisition. Structural T1-weighted images for spatial normalization were measured using a turbo field echo sequence with the following parameters: 274 sagittal slices covering the whole brain, flip angle = 8, 256 × 256 matrix, voxel size 0.7 × 0.7 × 0.7 mm^3^. For the resting state scans before and after stress induction (RS1 and RS2), 355 volumes of T2*-weighted echo-planar images were acquired for each session with the following parameters: 34 axial slices covering the whole brain, repetition time = 2000 ms, echo time = 30 ms, flip angle = 90, 9696  matrix, field of view = 240240 mm^2^, voxel size = 2.52.53 mm^3^.

### RS fMRI preprocessing

RS fMRI data were preprocessed and denoised in the CONN Functional Connectivity Toolbox v.18.a [49], a toolbox built upon the Statistical Parametric Mapping package (SPM12, Wellcome Centre for Human Neuroimaging) in MATLAB 2018 (The MathWorks, Inc.). The initial five volumes were removed from the data for T1 equilibration. The functional images were corrected for acquisition time differences between slices, then realigned to the first volume to correct for motion between volumes and resampled to 3 mm isotropic voxels. The anatomical images were resampled to match the functional images, then segmented into grey matter, white matter (WM), and cerebrospinal fluid (CSF). The co-registered functional images were normalized to Montréal Neurological Institute (MNI) space. Physiological noise was reduced by (1) regressing out five principal components of WM and CSF signal, and the 12 rigid body realignment parameters with CompCor, and (2) removing a first-order polynomial trend before bandpass-filtering the data to 0.01–0.1 Hz. Importantly, we did not perform global signal regression to avoid falsely increasing the anti-correlation between time series [50]. To account for head motion, outliers were identified using the implemented Artifact Detection Tools (ART) at an intermediate threshold. ART’s outlier detection is based on the calculation of three rigid body parameters in x, y, and z direction. A volume is labeled as an outlier if it contains 0.5 mm more motion than the previous volume or if the global mean signal intensity of the frame exceeds the mean intensity across all functional scans of a participant by three standard deviations. Participants/conditions were excluded whose sequence contained 30% or more outlier volumes or if any of the head motion parameters exceeded 3 mm in one of the four sessions (Nx4 RS1, Nx4 RS2, placebo RS1 and placebo RS2).

### Amygdala RSFC

Bilateral seeds were defined for the amygdala, according to probabilistic cytoarchitectonic maps defined by the Automated Anatomical Labeling (AAL) atlas [51]. To determine distinct seed regions with a high probability for the respective subregions, these maps were thresholded at $>80\%$ probability for the corresponding subregion [44]. The time course of the average preprocessed blood-oxygen-level-dependent (BOLD) signal within each region of interest (ROI) (left or right amygdala) was then correlated with signals in each voxel in the whole brain. Pearson correlation coefficients were then z-transformed (Fisher’s z), resulting in a matrix representing the voxel-wise strength of FC between the seed (left or right amygdala) and every voxel in the brain (zFC map). Finally, the zFC maps were smoothed using an 8 mm Full Width Half Maximum (FWHM) kernel before statistical analysis.

### Statistical analysis

To assess whether the effect of Nx4 on stress-induced Amygdala RSFC changes is modulated by trait anxiety levels, we first calculated a Nx4 efficacy measure as Nx4(RSFC2–RSFC1)—Placebo(RSFC2–RSFC1) for FC of left and right amygdala as seeds to every voxel in the brain. Next, we used voxel-wise multiple regression analysis, performed in SPM12, where correlations between Nx4 efficacy in the whole brain and anxiety level were calculated by adding TA as a covariate. Covariates to control for age and treatment sequence were added as well. Significant results from regression analysis were further examined: A linear mixed effects regression model (LMER) was built, explaining FC from the seed to the given resultant cluster based on treatment (placebo/Nx4) and session (RS1/RS2), with subject as a random intercept. Statistical analysis was performed using the lme4 package [52] in R. Post-hoc tests were applied with the emmeans package with Benjamini-Hochberg false discovery rate (FDR) method for correction of multiple comparisons [53]. Since changes due to stress induction are more evident in a sample with higher TA, we defined an “above average TA” subgroup based on a normative value of TA for working male adults (age range 19 to 69 years) which is suggested to be 34.9 [47]. Data from study participants with a TA score $\ge \ 35$ were reanalyzed for an Nx4 effect on stress-induced RSFC change as described above. We examined the RSFC changes after the psychosocial stress task in this above average TA subgroup and the Nx4 effect on these changes by LMER. Additionally, stress-induced changes of amygdala RSFC (contrast $RS2>RS1$) in placebo versus Nx4 conditions were compared by a paired t-test (RSFC2–RSFC1).

## Results

### Nx4 effect on stress-induced amygdala-prefrontal RSFC correlates with TA

**Fig. 3:**
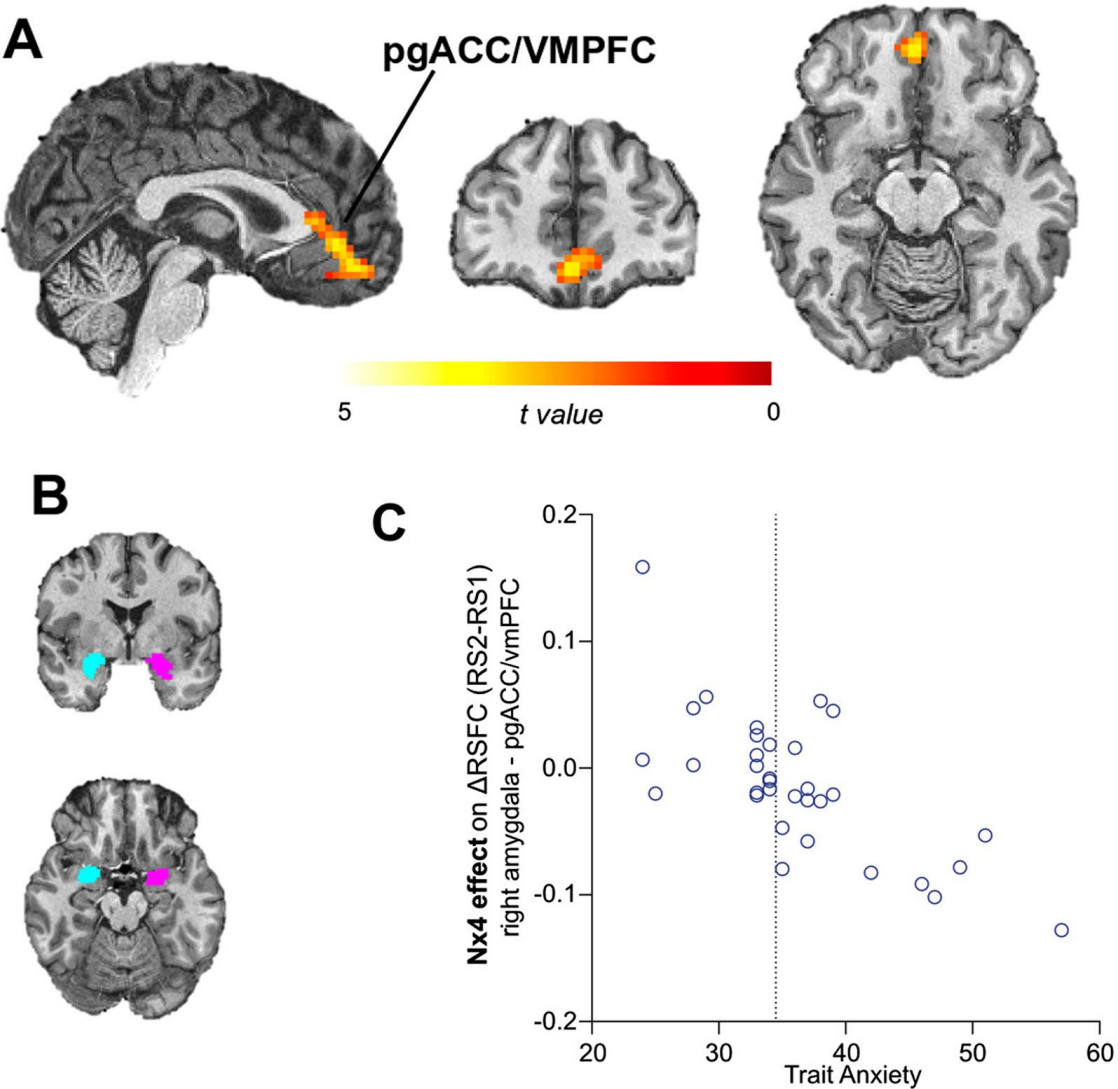
Trait anxiety (TA) correlates with Nx4 efficacy. On stress-induced changes in amygdala-centered resting state functional connectivity (RSFC). **A** Correlation of TA with Nx4 efficacy on RSFC changes from right amygdala showed a significant cluster in right amygdala and pregenual anterior cingulate cortex (pgACC)/ventro-medial prefrontal cortex (vmPFC) (p = 0.002; Family-Wise Error (FWE) corrected on cluster level). **B** Left and right amygdala seeds used to calculate amygdala functional network. **C** TA negatively correlated with Nx4 effects on stress-induced RSFC changes (contrast post-stress resting state (RS2) versus pre-stress resting state (RS1); $RS2>RS1$) from right amygdala to pgACC/vmPFC. The Nx4 effect takes placebo into account and is calculated as Nx4(RSFC2–RSFC1)—Placebo(RSFC2–RSFC1). Each dot in the scatter plot represents data from one participant. Normative average TA for the study population is indicated as a horizontal dashed line

Multiple linear regression analysis revealed that TA correlates with the Nx4 effect on stress-induced changes of amygdala RSFC. A significant cluster (peak t-value = 4.25; p = 0.002 cluster-level Family-Wise Error (FWE) corrected) was found for the right amygdala seed in the prefrontal cortex at MNI coordinates x = 0, y = 51, z = − 15 (Table 2). The region was identified as part of the pregenual anterior cingulate cortex (pgACC) and ventromedial prefrontal cortex (vmPFC) as shown in Fig. 3A and B. The resultant negative contrast of this association suggests that a higher TA score corresponds to a dampened stress-induced change of RSFC in the Nx4 compared to placebo condition, indicating a stronger Nx4 effect on stress response amelioration (Fig. 3C). For the left amygdala, no effect by TA level on the Nx4 effect was observed.

### TA correlates with stress-induced changes of amygdala-prefrontal RSFC

**Fig. 4:**
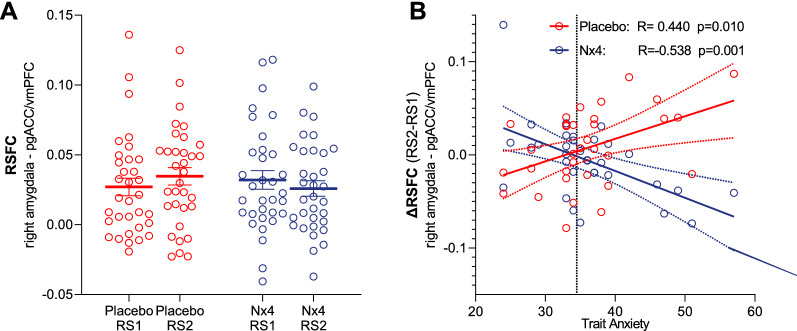
RSFC and TA correlate. Resting state functional connectivity(RSFC) between right amygdala and pregenual anterior cingulate cortex (pgACC)/ventro-medial prefrontal cortex (vmPFC) and its correlation with trait anxiety (TA) for placebo (red) and Nx4 (blue) condition for all 33 participants. **A** No significant differences were observed between pre-stress resting state (RS1) and post-stress resting state (RS2) for placebo nor for Nx4 conditions. Data are given as individual dot blots with meanstandard error of mean. **B** Stress-induced RSFC changes (contrast $RS2>RS1$) between right amygdala and pgACC/vmPFC is positively correlated with TA for placebo and negatively correlated with TA for Nx4 condition. Each dot in the scatter plot represents data from one participant. Dashed lines indicate 95% confidence interval of the linear model fit. Normative average TA for the study population is indicated as a horizontal dashed black line

In order to better understand the association of TA and Nx4 effect shown in the previous section, we conducted two analyses: First, TA scores were correlated to the stress-induced RSFC changes (stress contrast $RS2>RS1$ for the FC between amygdala and pgACC/vmPFC in the placebo condition only. For the placebo condition, we observed a significant positive correlation (R = 0.440; p = 0.010) between TA and the stress-induced RSFC changes from right amygdala to pgACC/vmPFC (Fig. 4B). This shows that participants with higher TA are more affected by the psychosocial stress induction for this particular stress response and show a greater increase in the right amygdala—pgACC/vmPFC FC after stress. Notably, in the Nx4 condition, the positive correlation between TA and RSFC changes was lost and we found a negative correlation between TA and stress-induced RSFC changes of right amygdala—pgACC/vmPFC in the Nx4 condition (R = − 0.539; p = 0.001, Fig. 4B) meaning that participants with higher TA show more pronounced RSFC decrease after treatment with Nx4.

In a second analysis, we only looked into pre- and post-stress RSFC between right amygdala and pgACC/vmPFC in placebo and Nx4 conditions separately (not including TA scores). We observed that this RSFC increased from RS1 (pre-stress) to RS2 (post-stress) for the placebo condition whereas it decreased under Nx4 (see Fig. 4A). However, none of these stress-induced RSFC changes reached a level of significance and no significant drug x time interaction effect was found (p = 0.268).

### Nx4 significantly reduced the amygdala-prefrontal RSFC in people with higher TA

**Fig. 5:**
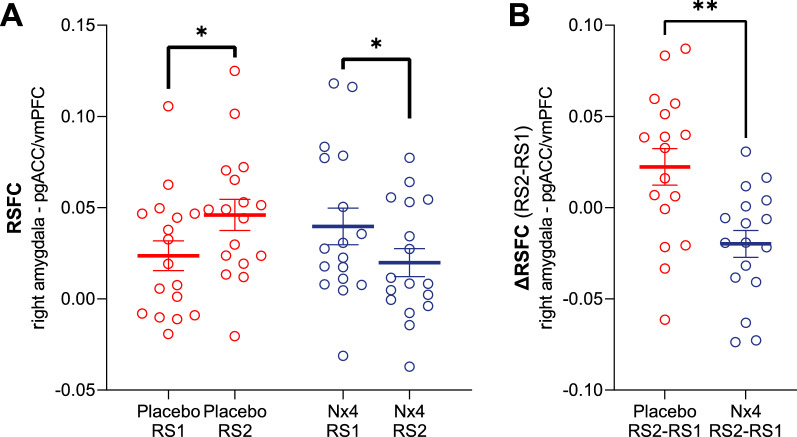
RSFC in high TA subgroup. Resting state functional connectivity (RSFC) between right amygdala and pregenual anterior cingulate cortex (pgACC)/ventro-medial prefrontal cortex (vmPFC) in the above average trait anxiety (TA) subgroup ($TA \ge \ 35$; n = 17) for placebo (red) and Nx4 (blue) condition. **A** RSFC increased from pre-stress resting state (RS1) to post-stress resting state (RS2) in placebo condition and decreased from RS1 to RS2 in Nx4 condition. **B** Stress-induced RSFC change (contrast $RS2>RS1$) is reduced in Nx4 versus placebo condition. Data are given as individual dot blots with meanstandard error of mean. Asterisks indicate significant differences (* $p<0.05$ and ** $p<0.01$)

The negative correlation between TA and Nx4 efficacy on stress-induced RSFC changes hints toward the fact that the efficacy of Nx4 on the stress-induced RSFC changes is greater for participants with higher TA. To demonstrate the Nx4 efficacy in a subgroup with more pronounced TA, we defined an above average TA subgroup based on normative values of TA for the study population [47]. For this above average TA subgroup, LMER model showed a significant Treatment x Time interaction (beta = − 1.15; 95% CI [− 1.85, − 0.46], p = 0.001), (Fig. 5A). Post hoc pairwise comparisons showed that RSFC between right amygdala and pgACC/vmPFC is increased significantly after stress for placebo (beta = − 0.02 [95% CI − 0.04 to − 0.004]; p = 0.01) whereas it decreased significantly in Nx4 condition (beta = 0.01 [95% CI 0.001 to 0.03]; p = 0.03). In addition, we compared stress-induced changes (contrast $RS2>RS1$) in placebo and Nx4 conditions by a paired t-test of the delta RFSC values (RSFC2–RSFC1). A significant reduction of stress-induced RSFC for the Nx4 condition was observed (t = 3.47; p = 0.003; Fig 5B).

## Discussion

In the present analysis, we used RS-fMRI before and after a stress-induction task to investigate the brain response at rest after an acute psychosocial stress induction. The effect of a single dose of Nx4 on the stress response, measured as amygdala centered RSFC, as well as the influence of TA on these neural mechanisms were evaluated. Regression analysis revealed a significant correlation between TA scores and Nx4 efficacy on stress-induced RSFC changes from right amygdala to prefrontal areas, centered in the vmPFC and pgACC. This suggests that a higher TA is associated with reduced stress-induced changes in Nx4 compared to placebo condition, indicating a stronger Nx4 effect. Additionally, we demonstrated a significant effect of Nx4 on stress-induced RSFC changes between right amygdala and pgACC/vmPFC in a subgroup of participants with above average TA levels.

### The amygdala-pgACC/vmPFC RSFC as a top-down inhibitory system

The regression analysis revealed a significant cluster in the pgACC/vmPFC for the right amygdala seed emphasizing a relevant role of the PFC in amygdala modulation. The FC between amygdala and vmPFC can be interpreted as a top-down inhibitory system controlled by vmPFC. This system is activated in healthy people during stress and emotional tasks [54, 55]. Malfunction of this system was reported in patients with mood and anxiety disorders or brain lesions who had a stronger amygdala response due to the lack of inhibitory modulation [23, 56–58]. The pgACC is known as a neural indicator of emotional control [59, 60], especially for regulating the amygdala and downstream endocrine responses during psychosocial stress [61]. Additionally, a correlation of increased cortisol levels and diminished pgACC-amygdala FC was described [62].

### Psychosocial stress increased the RSFC between amygdala and the pgACC/vmPFC in an above average TA subgroup

We observed an activation of this top-down inhibitory system after psychosocial stress induction, i.e. an increased RSFC between amygdala and pgACC/vmPFC in the placebo group. Very similarly, [27] showed an increased FC between amygdala and prefrontal regions in response to stressful stimuli as well. An increase in FC between amygdala and PFC was observed for emotion regulation after a stressor [63] as well as a positive correlation in FC of these areas with anxiety [64]. Accordingly, we could demonstrate an increase in FC after stress in placebo as well as a positive correlation of TA and the stress-induced changes in RSFC between amygdala and pgACC/vmPFC for the placebo condition, suggesting that participants with higher TA show a greater increase of amygdala-pgACC/vmPFC RSFC after stress. The stress-induced changes from RS1 to RS2 reached the level of significance only in the above average TA subgroup. This suggests a higher susceptibility to stress induction in this subgroup.

### Nx4 reduced the stress-induced changes in amygdala-pgACC/vmPFC RSFC

Whereas a positive correlation between stress-induced RSFC changes and TA was seen in the placebo condition, the intake of Nx4, reversed this relationship (Fig. 4B). Additionally, the above average TA subgroup experienced a significant increase of the RSFC from pre-stress to post-stress measurement under placebo whereas it decreased under Nx4 despite being exposed to the same stressful stimulus. In line with our proposition, a relative reduction of amygdala-prefrontal RSFC under Nx4 could mean that less down regulation of the amygdala by prefrontal areas is required under Nx4, as the amygdala is less activated by stress after Nx4 intake.

Reduced amygdala down regulation can be well related to anxiety. A recent study found a lesser extent of amygdala reactivity in the presence of emotionally negative stimuli in groups of people with explicitly low TA, i.e. expert meditators, compared to novices [64]. Under Nx4, the above average TA subgroup exhibited a response similar to what we would expect from a low-anxiety cohort. This could lead to the speculation that, firstly, Nx4 directly modifies amygdala reactivity and, secondly, has a particularly potent calming effect on anxious individuals. TA-dependent drug efficacy on this amygdala—PFC inhibitory system represents a plausible mechanistic hypothesis that seems worth testing in follow up studies.

### Lateralization: correlation of TA and changes in amygdala RSFC after stress only for right but not left amygdala

Interestingly, TA could only correlate changes in right amygdala RSFC after stress, whereas left amygdala RSFC remained non-significant. A similar lateralization effect in relation to TA was found for differential activation of the right amygdala for unconscious and conscious processing of fear [65]. This finding might be in accordance with the “right hemisphere hypothesis” postulating that emotions are predominantly processed in the right hemisphere [66]. Alternatively, one could hypothesize emotions were lateralized depending on their valence. According to the “valence hypothesis” the right hemisphere predominantly processes negative emotions and pain, whereas the left hemisphere is dominant for positive emotions [67, 68]. However, more recent meta-analyses found no support for the lateralization theory and rather suggested more left than right amygdala activation, particularly in response to negative emotional stimuli [69, 70].

Since our study only involved male participants, one could speculate that the lateralization of stress processing within the amygdala was a sex-specific effect. One of the first studies on such sex-effects demonstrated that right amygdala activation during encoding was related to enhanced emotional memory in men. In women, on the other hand, emotional memory could only be predicted by left amygdala activity [71]. In line with this, significant right-lateralization in the amygdala response were reported for male adolescents when viewing emotional faces, suggesting a lateralization even for simpler emotion recognition tasks [72]. Notably, [65] found a lateralization to the right hemisphere in a mixed sample. Furthermore, a meta-analysis could not identify any sex-specific lateralization effect in the amygdala in specific, although surrounding areas showed a significant male-right lateralization [70]. This leaves the possibility of particular emotional networks being differentially recruited in males and females, although there might not be a difference in amygdala activation per se.

Given that, to the best of our knowledge, no experimental parameter has been identified to predict hemispheric laterality [69, 70], we can only speculate about the origin of the right-lateralization found in this study.

### Limitations

The regression analysis was planned as an exploratory analysis intended for hypothesis generating to confine follow up investigations and does not make any confirmatory claims. In the regression analysis, the significant cluster in the PFC was found for the right amygdala seed but not for the left. Different studies report on lateralization of stress effects but to the best of our knowledge, no experimental parameter has been identified to robustly predict a hemispheric laterality. The definition of an above average TA subgroup was not contained in the initial hypothesis but introduced post hoc which compromises the statistical evidence of the outcome. Further, the number of participants in the subgroup was low and not balanced between placebo first and Nx4 first sequence. As described previously, the trial was limited to male participants with mild to moderate chronic stress. [41–45] and did not include clinically relevant outcome measures. Additionally the small sample size limits the generalizability of the present findings and results should be cautiously interpreted.

## Conclusion

We could show that a psychosocial stress task can lead to increased FC between the amygdala and the PFC in RS, after this task. The effect of the stress task on this RSFC is greater in anxiety prone subjects. Our data on Nx4, add evidence to the hypothesis that Nx4’s clinical efficacy is based on a dampened activation of the neural stress network and subjects with anxious personality traits might benefit more from Nx4 in terms of a reduction in their neuronal stress response. Further studies assessing clinically relevant outcome measures in parallel to fMRI are encouraged where behavioral aspects such as personality traits should be taken into consideration.

## Supplementary Information

**Additional file 1:** **Figure S1.**The ScanSTRESS task is composed of two runs, with alternating blocks of control and stress conditions of serial subtraction tasks and mental rotations. Control blocks did not contain any social evaluative feedback, time pressure or difficult questions whereas during stress blocks, participants were pushed for time, and two experimenters in professional attire explicitly showed their dissatisfaction with the correctness and speed of the answers via video stream. Task speed and difficulty were automatically adapted to the individual performance, ensuring that the participants were unable to meet the expectations. Between the two runs of the task, participants were interrupted and given extensive, disapproving verbal feedback.

**Additional file 2:** **Figure S2.**RSFC in high and low TA subgroup: Resting state functional connectivity (RSFC) between right amygdala and pregenual anterior cingulate cortex (pgACC)/ventro-medial prefrontal cortex (vmPFC) in the above average trait anxiety (TA) subgroup (upper row A and B) as well as in the below average TA subgroup (lower row C and D) for placebo (red) and Nx4 (blue) condition. (A) RSFC increased from pre-stress resting state (RS1) to post-stress resting state (RS2) in placebo condition and decreased from RS1 to RS2 in Nx4 condition. (B) Stress-induced RSFC change (contrast RS2>RS1) is reduced in Nx4 versus placebo condition. (C) No significant differences between RS1 and RS2 were observed for the below average TA subgroup. (D) No difference in Stress-induced RSFC change (contrast RS2>RS1) was observed between placebo and Nx4. Data are given as individual dot blots with mean±standard error of mean. Asterisks indicate significant differences (*p<0.05 and **p<0.01).

## Data Availability

The datasets presented in this article are not readily available because data belong to the sponsor of the clinical trial (Heel GmbH) and requires previous consent of the sponsor. Requests to access the datasets should be directed to martin.walter@med.uni-jena.de.

## Abbreviations

AAL: Automated Anatomical Labeling
AMST: Attention Modulation by Salience Task
ART: Artifact Detection Tools
BOLD: Blood-Oxygen-Level-Dependent (imaging)
CSF: Cerebrospinal fluid
EEG: Electroencephalography
FC: Functional connectivity
FDR: False Discovery Rate
FWE: Family-Wise Error
FWHM: Full Width Half Maximum
LMER: Linear mixed effects regression model
fMRI: functional Magnetic resonance imaging
HPA: Hypothalamic–Pituitary–Adrenal (axis)
MNI: Montreal Neurological Institute
PFC: Prefrontal Cortex
pgACC: pregenual Anterior Cingulate Cortex
PSS: Perceived Stress Scale
ROI: Region of interest
RS: Resting state
RS1: Resting state 1 (pre-stress)
RS2: Resting state 2 (post-stress)
RSFC: Resting state functional connectivity
RS-fMRI: Resting State functional Magnetic Resonance Imaging
SPM: Statistical Parametric Mapping
TA: Trait anxiety
TICS–SCSS: Trier Inventory for Chronic Stress–Screening Scale for Chronic Stress
vmPFC: ventromedial Prefrontal cortex
WM: White matter
