# Correction: Trait anxiety is related to Nx4’s efficacy on stress-induced changes in amygdala-centered resting state functional connectivity: a placebo-controlled cross-over trial in mildly to moderately stressed healthy volunteers

**DOI:** 10.1186/s12868-023-00776-6

**Published:** 2023-02-03

**Authors:** Melanni Nanni-Zepeda, Sarah Alizadeh, Tara Chand, Vanessa Kasties, Yan Fan, Johan van der Meer, Luisa Herrmann, Johannes C. Vester, Myron Schulz, Britta Naschold, Martin Walter

**Affiliations:** 1 Department of Psychiatry and Psychotherapy, Jena University Hospital, Philosophenweg 3, 07743 Jena, Germany; 2 Department of Psychiatry and Psychotherapy, University of Tubingen, Calwerstraße 14, 72076 Tübingen, Germany; 3 Leibniz Research Centre for Working Environment and Human Factors, Ardeystraße 67, 44139 Dortmund, Germany; 4 Department of Radiology and Nuclear Medicine, Amsterdam University Medical Center, Meibergdreef 9, 1105 AZ Amsterdam, Netherlands; 5 Idv Data Analysis and Study Planning, Tassilostraße 6, 82131 Gauting, Germany; 6 Biologische Heilmittel Heel GmbH, Dr.‑Reckeweg‑Str. 2‑4, 76532 Baden‑Baden, Germany

**Correction: BMC Neuroscience (2022) 23:68** 10.1186/s12868-022-00754-4

Following the publication of the original article [1], an error was identified in the **fMRI data acquisition** section.

The updated text is given below, and the changes have been highlighted in **bold typeface**.

**fMRI data acquisition**

A Philips 3T scanner was used for fMRI data acquisition. Structural T1-weighted images for spatial normalization were measured using a turbo field echo sequence with the following parameters: 274 sagittal slices covering the whole brain, flip angle = **8°**, 256 × 256 matrix, voxel size 0.7 × 0.7 × 0.7 mm^3^. For the resting state scans before and after stress induction (RS1 and RS2), 355 volumes of T2*-weighted echo-planar images were acquired for each session with the following parameters: 34 axial slices covering the whole brain, repetition time = 2000 ms, echo time = 30 ms, flip angle = **90°**, 9696 matrix, field of view = 240,240 mm^2^, voxel size = **2.5 × 2.5 × 3 mm**^**3**^.

Moreover, the authors identified an error in Table 1. The correct Table 1 is given in this correction.

**Table 1:** Demographics

	N	Age	TA	TICS	PSS
Whole group	33	43.1 ± 9.7	36.1 ± 7.4	15.7 ± 5.7	14.7 ± 3.7
High anxiety	17	40.6 ± 9.4	41.1 ± 6.5	17.1 ± 6.3	15.3 ± 4.6
Low anxiety	16	44.2 ± 10.2	30.7 ± 3.7	14.1 ± 4.6	14.0 ± 2.3

Summary statistics for number of participants (N), age in years, Trait Anxiety (TA), Trier Inventory for Chronic Stress (TICS) and perceived stress scale (PSS) are shown for the whole group as well as high and low anxiety subgroups. The low and high anxiety subgroups are defined based on the median of the TA score

Finally, the authors identified an error in Fig. 4. The correct Fig. 4 is given in this correction.

**Fig. 4:**
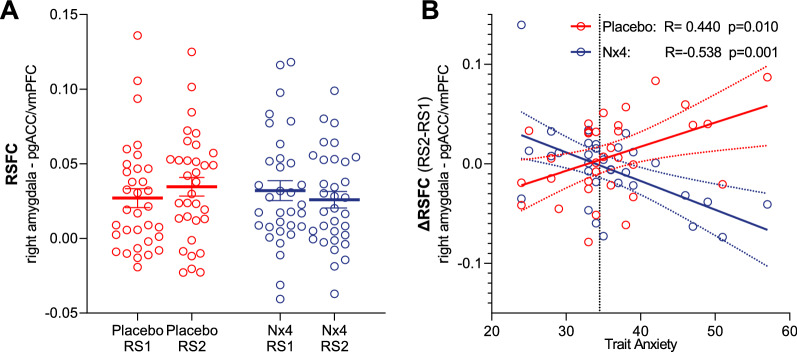
RSFC and TA correlate. Resting state functional connectivity (RSFC) between right amygdala and pregenual anterior cingulate cortex (pgACC)/ventro-medial prefrontal cortex (vmPFC) and its correlation with trait anxiety (TA) for placebo (red) and Nx4 (blue) condition for all 33 participants. **A** No significant differences were observed between pre-stress resting state (RS1) and post-stress resting state (RS2) for placebo nor for Nx4 conditions. Data are given as individual dot blots with meanstandard error of mean. **B** Stress-induced RSFC changes (contrast RS2 > RS1) between right amygdala and pgACC/vmPFC is positively correlated with TA for placebo and negatively correlated with TA for Nx4 condition. Each dot in the scatter plot represents data from one participant. Dashed lines indicate 95% confidence interval of the linear model fit. Normative average TA for the study population is indicated as a horizontal dashed black line

The original article [1] has been updated.
