# Supplementary material for: Trait anxiety is related to Nx4’s efficacy on stress-induced changes in amygdala-centered resting state functional connectivity: a placebo-controlled cross-over trial in mildly to moderately stressed healthy volunteers
Source: BMC Neurosci. 2022 Nov 24;23:68. doi: 10.1186/s12868-022-00754-4 (PMC9694608; doi:10.1186/s12868-022-00754-4)
Supplement: Supplementary file 1 — Additional file 1: Figure S1.The ScanSTRESS task is composed of two runs, with alternating blocks of control and stress conditions of serial subtraction tasks and mental rotations. Control blocks did not contain any social evaluative feedback, time pressure or difficult questions whereas during stress blocks, participants were pushed for time, and two experimenters in professional attire explicitly showed their dissatisfaction with the correctness and speed of the answers via video stream. Task speed and difficulty were automatically adapted to the individual performance, ensuring that the participants were unable to meet the expectations. Between the two runs of the task, participants were interrupted and given extensive, disapproving verbal feedback. [file 12868_2022_754_MOESM1_ESM.pdf]

Instructions	Announcing	Control Mental Rotation	Pause	Announcing	Control Subtraction	Pause	Announcing	Stress Mental Rotation	Pause	Announcing	Stress Subtraction	Pause	Announcing	Control Mental Rotation	Pause	Announcing	Control Subtraction	Pause	Announcing	Stress Mental Rotation	Pause	Announcing	Stress Subtraction
Verbal Feedback	Announcing	Control Mental Rotation	Pause	Announcing	Control Subtraction	Pause	Announcing	Stress Mental Rotation	Pause	Announcing	Stress Subtraction	Pause	Announcing	Control Mental Rotation	Pause	Announcing	Control Subtraction	Pause	Announcing	Stress Mental Rotation	Pause	Announcing	Stress Subtraction
Seconds	5	40	12.5	5	40	12.5	5	40	12.5	5	40	12.5	5	40	12.5	5	40	12.5	5	40	12.5	5	40
Minutes	7.5																						

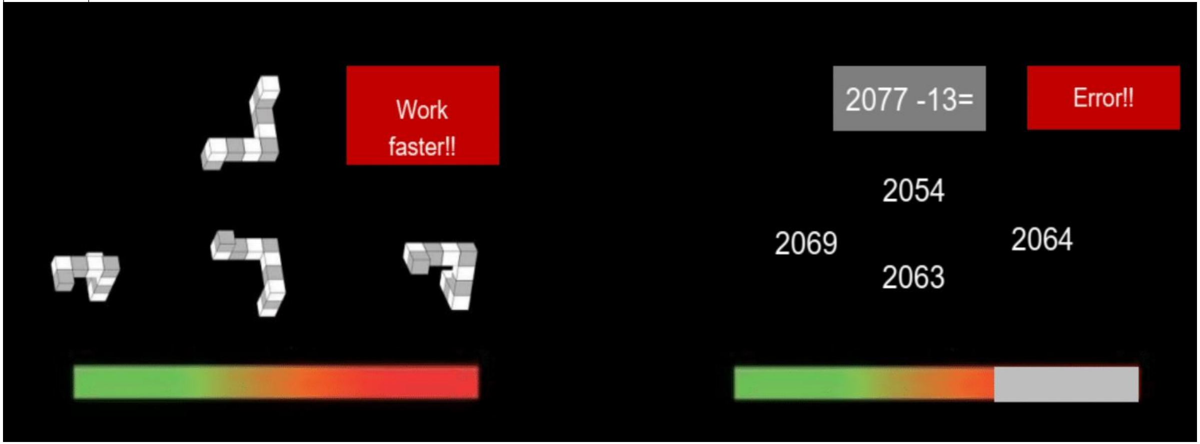

Supplementary Figure S1: The ScanSTRESS task is composed of two runs, with alternating blocks of control and stress conditions of serial subtraction tasks and mental rotations. Control blocks did not contain any social evaluative feedback, time pressure or difficult questions whereas during stress blocks, participants were pushed for time, and two experimenters in professional attire explicitly showed their dissatisfaction with the correctness and speed of the answers via video stream. Task speed and difficulty were automatically adapted to the individual performance, ensuring that the participants were unable to meet the expectations. Between the two runs of the task, participants were interrupted and given extensive, disapproving verbal feedback.
