# Supplementary material for: Trait anxiety is related to Nx4’s efficacy on stress-induced changes in amygdala-centered resting state functional connectivity: a placebo-controlled cross-over trial in mildly to moderately stressed healthy volunteers
Source: BMC Neurosci. 2022 Nov 24;23:68. doi: 10.1186/s12868-022-00754-4 (PMC9694608; doi:10.1186/s12868-022-00754-4)
Supplement: Supplementary file 2 — Additional file 2: Figure S2.RSFC in high and low TA subgroup: Resting state functional connectivity (RSFC) between right amygdala and pregenual anterior cingulate cortex (pgACC)/ventro-medial prefrontal cortex (vmPFC) in the above average trait anxiety (TA) subgroup (upper row A and B) as well as in the below average TA subgroup (lower row C and D) for placebo (red) and Nx4 (blue) condition. (A) RSFC increased from pre-stress resting state (RS1) to post-stress resting state (RS2) in placebo condition and decreased from RS1 to RS2 in Nx4 condition. (B) Stress-induced RSFC change (contrast RS2>RS1) is reduced in Nx4 versus placebo condition. (C) No significant differences between RS1 and RS2 were observed for the below average TA subgroup. (D) No difference in Stress-induced RSFC change (contrast RS2>RS1) was observed between placebo and Nx4. Data are given as individual dot blots with mean±standard error of mean. Asterisks indicate significant differences (*p<0.05 and **p<0.01). [file 12868_2022_754_MOESM2_ESM.pdf]

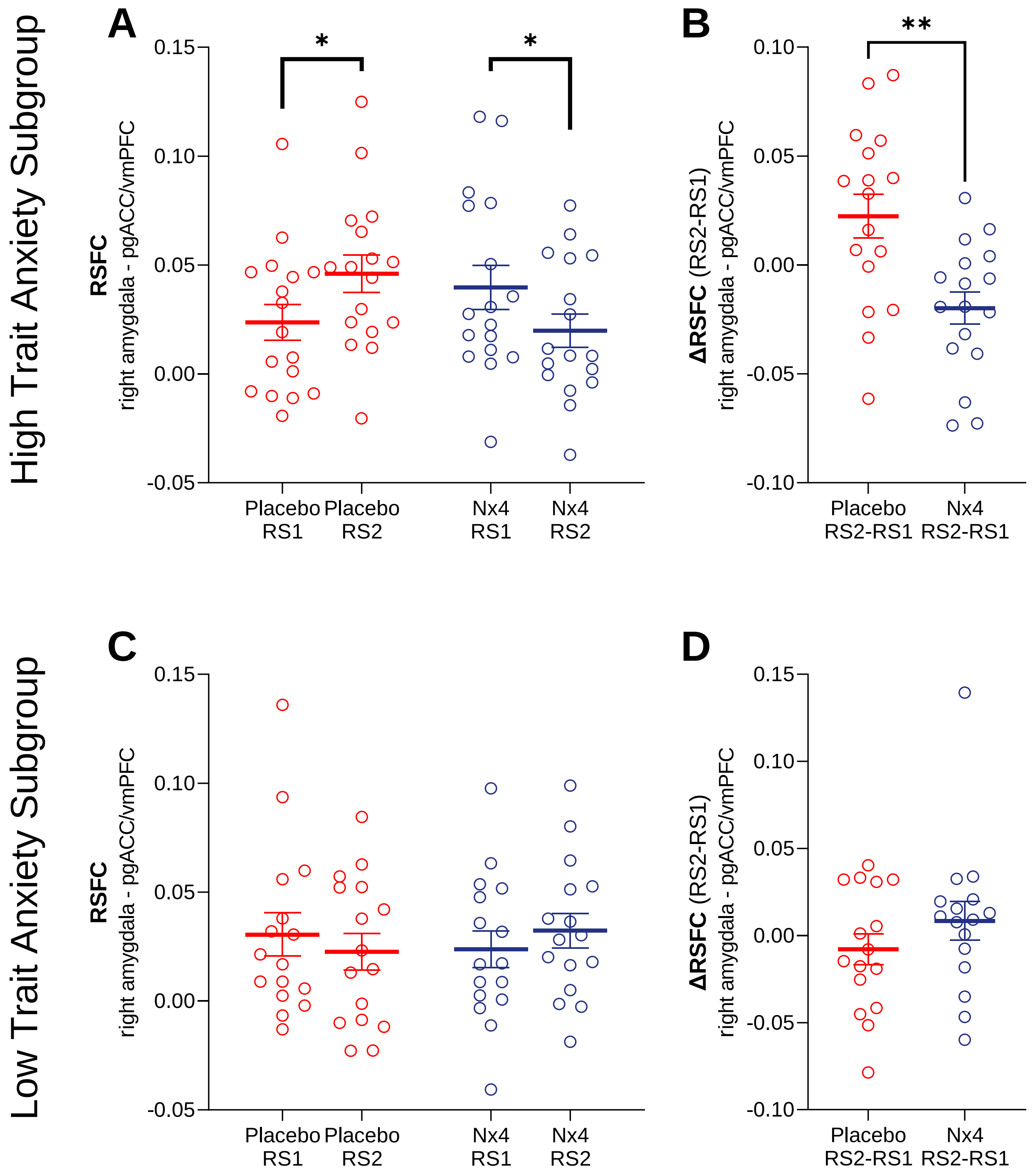


**Supplementary Figure S2 - RSFC in high and low TA subgroup**: Resting state functional connectivity (RSFC) between right amygdala and pregenual anterior cingulate cortex (pgACC)/ventro-medial prefrontal cortex (vmPFC) in the above average trait anxiety (TA) subgroup (upper row A and B) as well as in the below average TA subgroup (lower row C and D) for placebo (red) and Nx4 (blue) condition. (**A**) RSFC increased from pre-stress resting state (RS1) to post-stress resting state (RS2) in placebo condition and decreased from RS1 to RS2 in Nx4 condition. (**B**) Stress-induced RSFC change (contrast RS2>RS1) is reduced in Nx4 versus placebo condition. (**C**) No significant differences between RS1 and RS2 were observed for the below average TA subgroup. (**D**) No difference in Stress-induced RSFC change (contrast RS2>RS1) was observed between placebo and Nx4. Data are given as individual dot blots with mean±standard error of mean. Asterisks indicate significant differences (*p<0.05 and **p<0.01).}
